# How to engage Cofilin

**DOI:** 10.1186/1742-4690-5-85

**Published:** 2008-09-22

**Authors:** Michael Bukrinsky

**Affiliations:** 1 The George Washington University, Department of Microbiology, Immunology and Tropical Medicine, Washington, DC 20037, USA

## Abstract

In HIV-infected people, resting CD4+ T cells are the main reservoir of latent virus and the reason for the failure of drug therapy to cure HIV infection. Still, we do not have a complete understanding of the factors regulating HIV replication in these cells. A recent paper in *Cell *describes a new trick that the virus uses to infect resting T cells. Interaction between the viral gp120 and cellular HIV co-receptor, CXCR4, during viral entry initiates signaling that activates cofilin, the main regulator of actin polymerization. As a result of this activation, actin is depolymerized, thus destroying the natural barrier to HIV replication. I discuss implications of this study for our understanding of HIV biology and development of novel anti-HIV therapeutic approaches.

HIV accesses target cells via interaction between the viral envelope protein, gp120, and viral receptor and co-receptor, CD4 and CCR5 or CXCR4, respectively, on infected cells [1]. While interaction of gp120 with CD4, CCR5 or CXCR4 has been shown to induce intracellular signaling events [2-4], the role of this signaling in HIV replication remains controversial. Initial reports suggested that signaling from chemokine co-receptor is not essential for HIV-1 infection [5-7]. Indeed, transfection of CD4-positive CCR5-negative cells with mutant CCR5 unable to transduce signals made such cells fully susceptible to infection with R5 HIV-1 (R5 viruses infect cells expressing CD4 and CCR5, while X4 viruses infect cells with CD4 and CXCR4). However, these results were obtained using transformed T cell lines. In contrast, HIV-1 infection of activated primary CD4^+ ^T cells, the main viral target in the body, appears to depend on signaling mediated by chemokine co-receptors [8-10]. The mechanism of this effect was found to involve actin-dependent re-localization of HIV receptors resulting in co-capping of CD4 and chemokine co-receptor [8,10], which stimulates HIV entry.

A recent paper by Wu and colleagues [11] analyzed the role of signaling in HIV infection of another important viral target, resting CD4^+ ^T cells. These cells, which form the main reservoir of latent virus in the body [12], are relatively resistant to HIV infection, as reverse transcription and nuclear transport of the viral pre-integration complex (PIC) are very inefficient in non-activated T cells [13-15]. However, robust viral replication can be induced by activation of HIV-inoculated resting T cells, even several days after infection [14,16]. The authors used this model to demonstrate that pre-treatment of cells with pertussis toxin (PTX), which uncouples chemokine receptor from G proteins thus inhibiting signaling, greatly reduced HIV replication when cells were subsequently activated via CD3/CD28 stimulation [11]. In contrast, no such decrease was observed when cells were pre-treated with damnacanthal, which inhibits Lck, a signaling molecule coupled to CD4. Given that the HIV-1 NL4-3 virus used for infection is CXCR4-tropic, these results indicate that signaling from the CXCR4 receptor, but not from CD4, is critical for the ability of HIV to establish latent infection of non-activated T cells.

In an attempt to identify the step in viral replication controlled by CXCR4 signaling, the authors analyzed the early steps of infection, starting from fusion to integration. This analysis revealed that, while fusion and reverse transcription were not affected by PTX pre-treatment and thus not dependent on CXCR4-originating signaling, nuclear translocation of the viral DNA was inhibited [11]. Therefore, similar to T cell activation by PHA or CD3/CD28 stimulation [17], CXCR4 signaling activates nuclear translocation of the viral pre-integration complex, however, it does not activate reverse transcription, another critical event in the re-activation of latent HIV infection of resting T cells [13]. Of note, in contrast to CD3/CD28 activation, stimulation of HIV nuclear translocation by CXCR4 signaling was small (about 3-fold relative to PTX-pre-treated cells), and a real difference was observed only after cells were activated by CD3/CD28. Therefore, CXCR4 signaling appears to facilitate the movement of the HIV-1 genome towards and into the nucleus poising it for cell activation. When this signaling is blocked, the viral genome becomes supposedly localized to a compartment where it cannot be activated, and then would undergo eventual degradation.

So what is the nature of this compartment? An intriguing observation reported by Yoder et al. is that HIV-induced CXCR4-dependent signaling triggers rapid polymerization and subsequent depolymerization of the cortical actin filaments (Fig. 1). Actin polymerization is an essential mechanism of chemotactic cell motility induced by chemokines [18], and has been documented for CXCR4-dependent T cell chemotaxis in response to SDF-1 or gp120 stimulation [19,20]. An unexpected finding by Yoder et al. is HIV-induced rapid depolymerization of polymerized actin (F-actin). When actin depolymerization was blocked by actin-stabilizing agent. jasplakinolide, HIV replication following cell activation was inhibited. This result suggests that actin depolymerization is critical for HIV replication. Previously, association of incoming HIV with F-actin was proposed to be a necessary step in formation of the reverse transcription complex and reverse transcription [21]. It now appears that this association has to be very transient, and if not disrupted within 5 minutes after infection by actin depolymerization, it will prevent subsequent steps of HIV replication. It remains to be determined what happens to the viral reverse transcription complex during the first 5 minutes after entry and why this first time period is so critical for subsequent replication.

**Figure 1:**
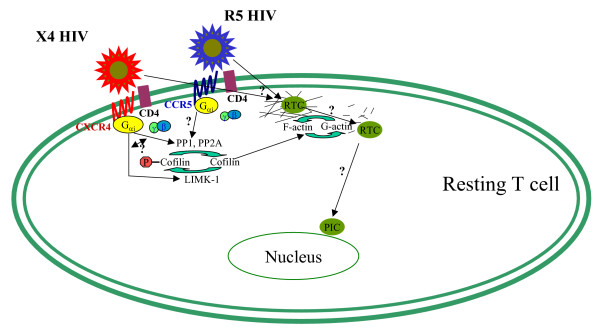
**A model depicting actin regulation by cofilin during HIV-1 infection of resting T cell**. Interactions between the key factors involved in regulation of cortical actin are shown in the context of HIV-1 infection. HIV-induced signaling from CXCR4 activates phosphatase which dephosphorylates and activates cofilin. This leads to depolymerization of F-actin, releasing HIV-1 reverse transcription complex and promoting its translocation towards the nucleus. Steps requiring additional studies, such as involvement of CCR5 in cofilin activation, regulation of a switch between activation of cofilin kinase and phosphatase, the role of F-actin in HIV reverse transcription and nuclear translocation are marked by question marks. See text for details.

Thus, HIV-induced CXCR4 signaling depolymerization of cortical actin appears to support viral replication. A similar mechanism was previously ascribed to HIV-1 protein Nef, which can disrupt actin cytoskeleton and promote HIV replication [22,23]. Such multifaceted effort by the virus to depolymerize actin implies a critical role of this process in viral infection. A number of important questions remain to be addressed before this model can be accepted conclusively. Is the association with F-actin necessary for reverse transcription? Given the rapid depolymerization of actin in HIV-infected cells, this would seem unlikely. What is the role of F-actin in nuclear translocation of HIV pre-integration complex (PIC)? Live microscopy has shown that most viral particles in the cytoplasm are associated with microtubules and not with F-actin [24]. The majority of particles that associated with actin were found in the peripheral regions of the cytoplasm. This finding is consistent with rapid transfer of the PIC from actin to tubulin, which may be promoted by depolymerization of actin.

The key regulator of actin assembly and disassembly is cofilin, a member of the actin-depolarizing factor (ADF) family of proteins [25]. Cofilin's association with actin promotes depolymerization of actin filaments [26]. Association of cofilin with actin is regulated by phosphorylation: cofilin phosphorylation at serine 3 by LIM kinase 1 prevents its association with actin [27], whereas dephosphorylation by phosphatases PP1 and PP2A stimulates this association and actin depolymerization [28]. Yoder et al. reported that in resting T cells, cofilin was largely phosphorylated (inactive), but was activated by dephosphorylation within minutes after HIV infection [11]. In contrast, cofilin in CD3/CD28-activated T cells or transformed T cell lines is in a permanently activated state. Interestingly, a transient increase in cofilin phosphorylation was observed immediately after HIV infection. This kinetic is consistent with a model that predicts a rapid association of the viral complex with F-actin which is required to form a functional reverse transcription complex (RTC), followed by depolymerization of F-actin, RTC relocation to microtubules, and migration toward the nucleus [29]. In support of this model, downregulation of cofilin using shRNA led to increased reverse transcription but decreased nuclear translocation [11].

In summary, the paper by Yoder et al. reveals a new factor that regulates HIV infection of resting T cells, and suggests new approaches to anti-HIV therapeutic interventions. However, before treatments targeting F-actin could be developed as anti-HIV therapy, several critical questions remain to be addressed (see Fig. 1). Is the observed phenomenon specific for CXCR4 or is it also true for CCR5 signaling? Given that both receptors are coupled to G_αi_, one would expect that CCR5 signaling should also activate cofilin. And what is the state of cofilin activation in various populations of T cells, in particular non-activated memory T cells which are the main targets of HIV infection? In fact, T cells in the body exist in various states of activation, from fully quiescent to fully activated, as reflected by the expression of various activation markers [30]. It would be important to correlate the state of cellular activation with the status of cofilin activation, and ideally identify a cell surface marker indicative of cofilin activation. A number of mechanistic questions also beg for attention. How is CXCR4 signaling transduced to cofilin? How is the switch between initial inactivation (phosphorylation) and subsequent activation (dephosphorylation) of cofilin regulated? What happens to the virus during the time between the entry into a resting cell and subsequent cell activation? How does actin depolymerization stimulate nuclear translocation of the viral PIC? It appears that a new exciting field of studies has been born which will greatly advance our knowledge of HIV biology.
